# Primary squamous cell carcinoma of renal parenchyma: A case report and literature review

**DOI:** 10.3389/fonc.2023.1037156

**Published:** 2023-03-30

**Authors:** Ke Liang, Yichu Yuan, Bodong Lv, Zunjin Ke

**Affiliations:** ^1^ Department of Urology, The No. 1 People’s Hospital of Pinghu, Pinghu, China; ^2^ Department of Urology, The Second Affiliated Hospital of Zhejiang University School of Medicine, Hangzhou, China

**Keywords:** carcinoma, squamous cell carcinoma, renal parenchyma, pathology, prognosis

## Abstract

**Rationale:**

Primary renal parenchymal squamous cell carcinoma (SCC) is an extremely rare tumor that is difficult to diagnose by hematology and imaging studies and is often diagnosed later than other primary renal cancers.

**Diagnosis:**

A 52-year-old male patient was found to have cysts in both kidneys for 1 week. No urgency and frequency of urination, no dysuria, no gross hematuria, and no significant changes in recent body weight were reported.

**Interventions:**

The upper pole of the right kidney is a cystic and solid mass (8.3 cm * 8.2 cm * 8.1 cm), the cystic part has long T1 and long T2 signals, the solid part has mixed signals, and some parts have limited diffusion. There were nodular long T1 and short T2 calcification signals. An enhanced scan of the solid part showed uneven enhancement and continuous enhancement of the mass capsule. Cystic renal cancer was considered because of the multiple cysts in both kidneys. Surgical treatment was performed. Postoperative pathology revealed well-differentiated squamous cell carcinoma of the right kidney with cystic degeneration, 8.5 cm * 6 cm in size, infiltrating the renal parenchyma, and the cutting edge was negative. The pathological stage was pT2bN0M0.

**Outcome:**

At the follow-up 5 months after the operation, no metastasis was found.

**Conclusion:**

Renal SCC is rare and easily misdiagnosed and missed. Pathological diagnosis is still the gold standard for its diagnosis. However, with active surgical treatment, the short-term prognosis of the patient is good.

## 1 Introduction

Primary renal parenchymal squamous cell carcinoma (SCC) is an extremely rare tumor that is difficult to diagnose by hematology and imaging studies and is often diagnosed later than other primary renal cancers. This article reports a case of squamous cell carcinoma originating in the renal parenchyma. After active surgical treatment, the postoperative recovery was good, but the prognosis still needs long-term follow-up.

## 2 Case presentation

This human study was approved by The Second Affiliated Hospital of Zhejiang University School of Medicine Institutional Review Board, and the patient provided informed consent for the publication of the case.

A 52-year-old male patient was admitted to the hospital on 4 January 2022 for “one week of renal cyst found in physical examination”. One week prior, the patient went to the hospital for a physical examination. Abdominal B-ultrasound showed that there were multiple cysts in both kidneys, including a larger right kidney with calcification of the cyst wall and multiple calcifications. Further enhanced CT scan of the kidney revealed cystic solid lesions of the right kidney with multiple calcifications. There was no acute urination, no dysuria, and no gross hematuria. There had been no recent significant change in body weight. Upon physical examination, no obvious mass was found in the waist and abdomen, and there was no percussion pain in the renal region. There was no abnormality in routine hematuria and renal function. The tumor markers were as follows: squamous cell carcinoma antigen (SCCA) 4.1 μg/ml, alpha-fetoprotein (AFP) 3.6 ng/ml, carcinoembryonic antigen (CEA) 3.3 ng/ml, renal contrast-enhanced MR revealing a cystic solid mass in the right upper pole (8.3 cm * 8.2 cm * 8.1 cm), the signal of the solid part was mixed, the solid part was inhomogeneously enhanced, and the capsule of mass was continuously enhanced. Cystic renal carcinoma and multiple renal cysts were considered. Emission computed tomography (Ect) examination: mild-to-moderate reduction of left renal function and a right renal cyst. Right upper pole renal cell carcinoma was considered. On 6 January 2022, a robot-assisted partial nephrectomy was performed under general anesthesia. The patient was placed in the left lateral position, a channel was established in the posterior peritoneum, and the extraperitoneal fat and perirenal fascia were cleaned. It was found that the tumor was located in the upper pole of the kidney, protruding from the surface of the kidney, approximately 8 cm * 8 cm in size, was cystic solid, and had no obvious adhesion to the surrounding area. The tumor was completely resected with scissors along the surface of the tumor, 0.5 cm away from the edge of the tumor. The gross specimen revealed well-differentiated squamous cell carcinoma of the right kidney with cystic change, 8.5 cm * 6 cm in size, infiltrating the renal parenchyma, with the negative cutting edge of the broken end. Pathological diagnosis was pT2bN0M0. Immunohistochemistry showed Pax-8 (+), CK5/6 (+), P40 (+), p63 (+), and GATA-3 (partial +). Gene detection revealed that there was no first-order variation with clear clinical significance and nine secondary variants with potential clinical significance (ARID1A p.P1207fs, FAT1 p.Q961X, FBXW7 p.S92X, FGFR3 p.S249C, KMT2D p.L4152fs, KMT2D p.P4324fs, NFE2L2 p.L30F, PIK3CA p.E545K, and TERT c.-124G>A). Five months after the operation, a CT examination showed no local or retroperitoneal metastasis. Tumor markers were as follows: SCCA 0.8 μg/ml, AFP 2.0 ng/ml, and CEA 1.2 ng/ml.

## 3 Discussion

Primary squamous cell carcinoma of the kidney is a rare urological tumor, accounting for 0.5%–0.8% of renal malignancies (1), including renal pelvis SCC and renal SCC. There are more and more reports on renal pelvis SCC in clinical practice. Most experts believe that long-term renal pelvic stones, chronic pelvic inflammatory reaction, and hydronephrosis are the main reasons for inducing SCC (2–4). There are also reports that chemical substances (including drug abuse), hormone imbalance in the body, vitamin A deficiency, and chronic inflammatory reactions of renal transplantation are all potential causes (5). With the deepening of research, renal pelvis SCC is considered to be caused by urothelial metaplasia (6). Primary renal parenchyma SCC is extremely rare. So far, only nine cases including this case have been reported (2–4, 6–10) **Table 1**. The mechanism of the disease is not clear.

**Table 1:** Case review.

	Author	Age (years)	Presentation	Location	Treatment	Treatment size	Tumor extent	New targeted drugs	chemotherapy	Prognosis
1	Terada (8)	73	Hematuria and lumbago	Multiple: bladder, left ureter, and left kidney	Cystectomy and nephroureterectomy	NA, with perirenal fat invasion	pT4N1M1	Absent	Absent	Alive and disease free at 3 months after surgery
2	Kulshreshtha (9)	60	Weight loss for 3 months	Mid and lower pole of the left kidney	Radical nephrectomy with lymph node dissection	6.5 × 5.5 cm	pT4N1M0	Absent	Absent	Alive and disease free at 13 months after surgery
3	Ghosh (2)	51	Dull and intermittent flank pain for 5 months	Lower pole of the right kidney	Radical nephrectomy	5.8 × 5.5 cm	pT1bN0M0	Absent	Absent	Alive and disease free at 12 months after surgery
4	Sahoo (6)	50	Right abdomen pain for 6 months	Upper pole of the right	Radical nephrectomy	8.0 × 6.0 cm	pT2aNxM0	Absent	Absent	Alive and disease free at 6 months after surgery
5	Wang (3)	61	Hematuria and lumbago for 2 months	Right kidney	Radical nephrectomy	NA, with perirenal fat invasion	pT3aNxM0	Absent	Absent	Alive and disease free at 1 month after surgery
6	Zhang (4)	61	Intermittent flank pain for 2 months	Lower pole of the right kidney	Radical nephrectomy	NA, with perirenal fat invasion	pT3aNxM0	Absent	Absent	Alive and disease free at 3 months after surgery
7	Fotovat (7)	41	Flank pain and dysuria for 3 months	Lower pole of the left kidney	Radical nephrectomy	NA, with perirenal fat invasion	pT3aN1M0	Absent	Present	Metastasis to the ovary at 8 months after surgery
8	Present (2022)	61	Flank pain and weight loss for 2 months	Lower pole of the right kidney	Radical nephrectomy with right hemicolectomy	9.0 × 8.0 cm	pT4N0M0	Absent	Absent	Alive and disease free at 5 months after surgery
9	Liang	52	1 week of renal cyst found in physical examination	Upper pole of the right kidney	Robot-assisted partial nephrectomy	8.3 × 8.2 × 8.1 cm	pT2aNxM0	Absent	Absent	Alive and disease free at 6 months after surgery

The onset of primary renal SCC is insidious, with no obvious symptoms, typical signs, and imaging manifestations in the early stage. It is often covered by hydronephrosis, calculus, or inflammation, which is easy to be misdiagnosed and missed. Compared with other types of tumors, it is often diagnosed later (11). According to the reported primary renal SCC, 88.89% (8/9) patients had lumbar and abdominal pain, and 22.22% (2/9) had hematuria. There was no significant difference in the incidence rate between sexes (5 men and 4 women). The most common tumors were on the right side (6/9 cases) and the inferior pole (5/9 cases). Although two cases were associated with renal calculi, the histology of the renal pelvis system was normal in all cases. More than half of the cases (5/9) were in the advanced stage (higher than pT3), and two cases had lymph node metastasis, which led to a worse prognosis. The patient was diagnosed with a renal cyst upon physical examination, and the tumor did not invade the collection system. FDG-PET/CT result was improved, and the possibility of metastatic renal SCC was excluded. However, FDG-PET/CT was not effective in distinguishing renal inflammatory diseases from various types of tumors (3). SCCA is a tumor-associated antigen extracted from cervical squamous cell carcinoma. It has certain clinical value in the diagnosis and monitoring of tumor recurrence of squamous cell carcinoma (12). With textual research considering the non-specific characteristics of the case, it is difficult to make a definite diagnosis before the operation. For imaging findings of renal tumors, a biopsy can be performed to confirm the diagnosis. Ureteroscopy and urine cytology are helpful in the diagnosis of renal pelvis SCC or invasive lesions.

For primary renal SCC, a pathological examination is still the gold standard for diagnosis. As it is not sensitive to chemoradiotherapy, radical nephrectomy is recommended for treatment, and surgery is also the most effective way for patients with local metastasis. All reported cases were followed up for an average of 4.22 months, and all patients survived. Only one pT3aN1 patient had ovarian metastasis (7). The patient was followed up for 0.5 years. There were no signs of recurrence and metastasis, indicating that early diagnosis and early treatment of renal SCC can achieve a better curative effect, but it is still lacking long-term follow-up verification. If the tumor relapses, there are still many targeted drugs to choose from according to the results of gene detection. Examples include bevacizumab combined with everolimus (T3A), sunitinib (T3A), temsirolimus (T3B), and mTOR inhibitors (sirolimus, everolimus, and T4) (based on the patient’s gene detection).

## 4 Conclusion

Primary renal parenchymal SCC is a very rare tumor, and at hematological and imaging examinations, diagnosis is difficult, compared with other primary renal cell carcinomas, and its diagnosis is often late. There is still no unified treatment plan, surgery is still the main choice, and new targeted drugs also provide individualized schemes, but their efficacy still lacks clinical follow-up verification.

## Data availability statement

The original contributions presented in the study are included in the article/**Supplementary Materials**. Further inquiries can be directed to the corresponding author.

## Ethics statement

The studies involving human participants were reviewed and approved by the Ethics Committee of the First People’s Hospital of Pinghu City. The patients/participants provided their written informed consent to participate in this study. Written informed consent was obtained from the individual(s) for the publication of any potentially identifiable images or data included in this article.

## Author contributions

Conceptualization: KL. Investigation: KL. Supervision: YY. Writing—original draft: KL. Formal analysis: BL. Funding support: ZK. All authors contributed to the article and approved the submitted version.
