# Supplementary material for: Primary squamous cell carcinoma of renal parenchyma: A case report and literature review
Source: Front Oncol. 2023 Mar 30;13:1037156. doi: 10.3389/fonc.2023.1037156 (PMC10098210; doi:10.3389/fonc.2023.1037156)
Supplement: Supplementary file 1 [file DataSheet_1.docx]

Supplementary Material

##
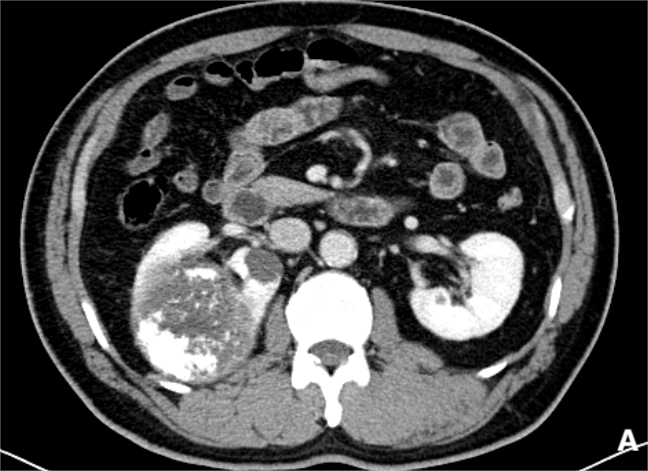

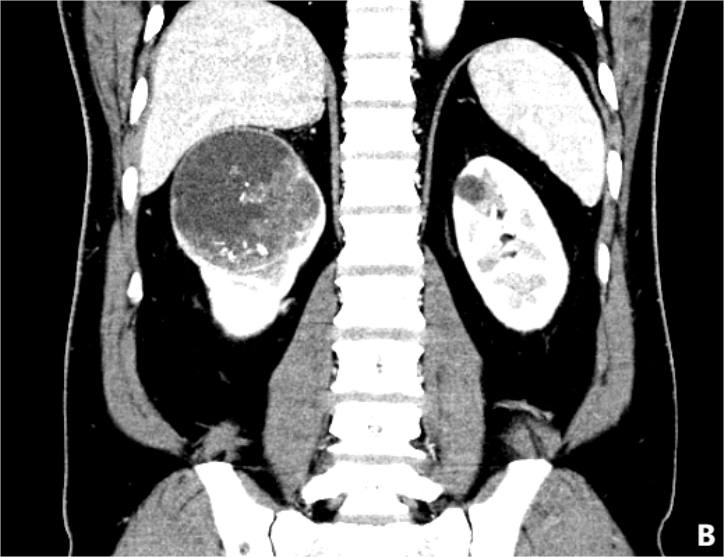
1 Supplementary Figures


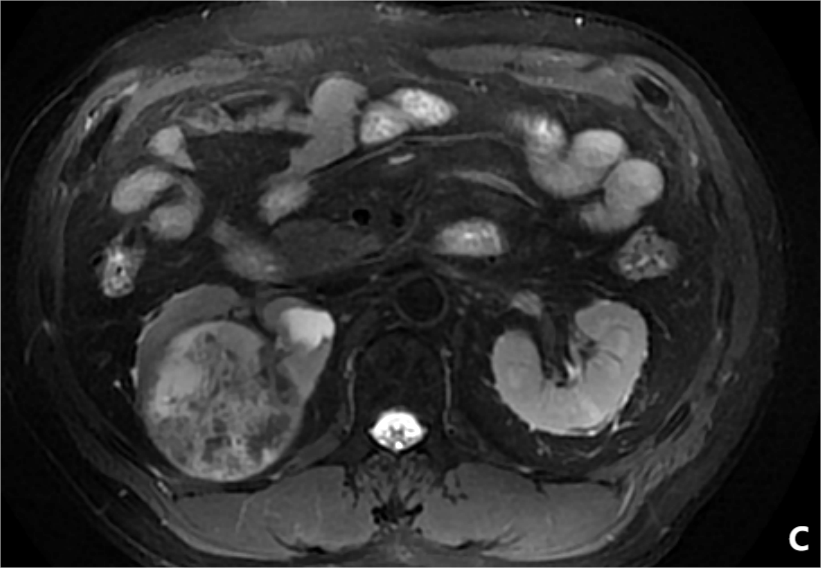

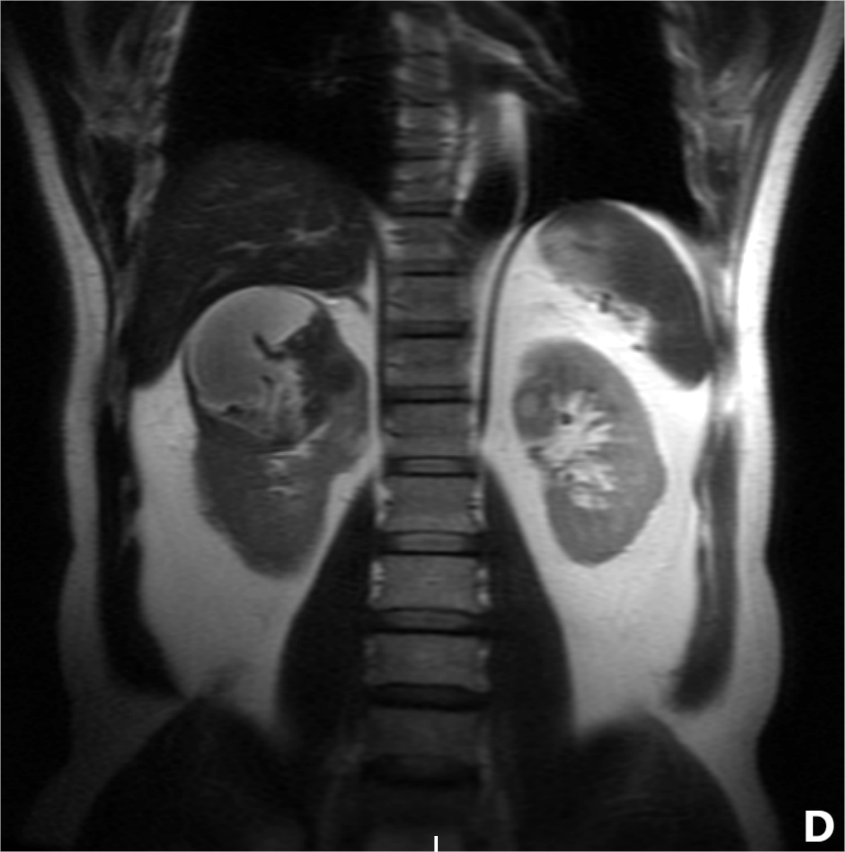


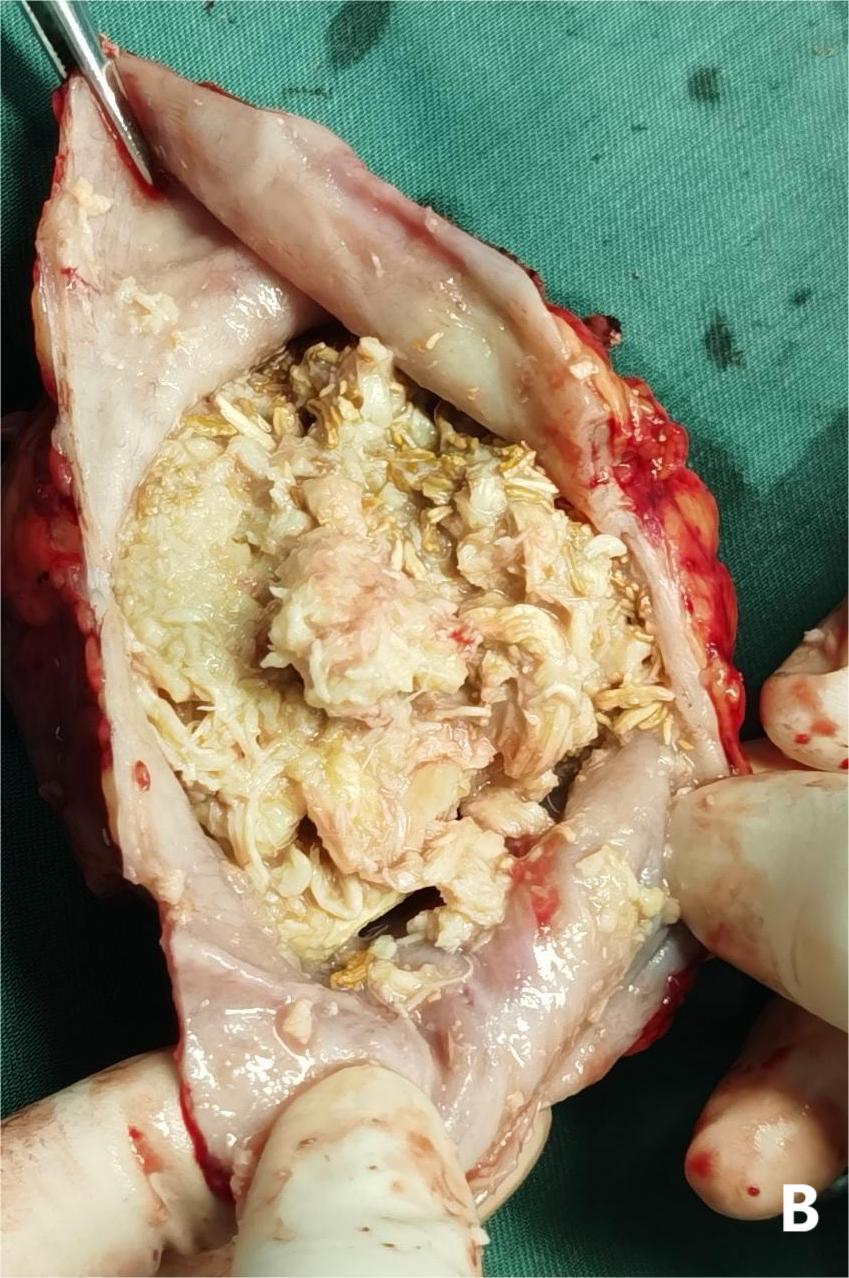

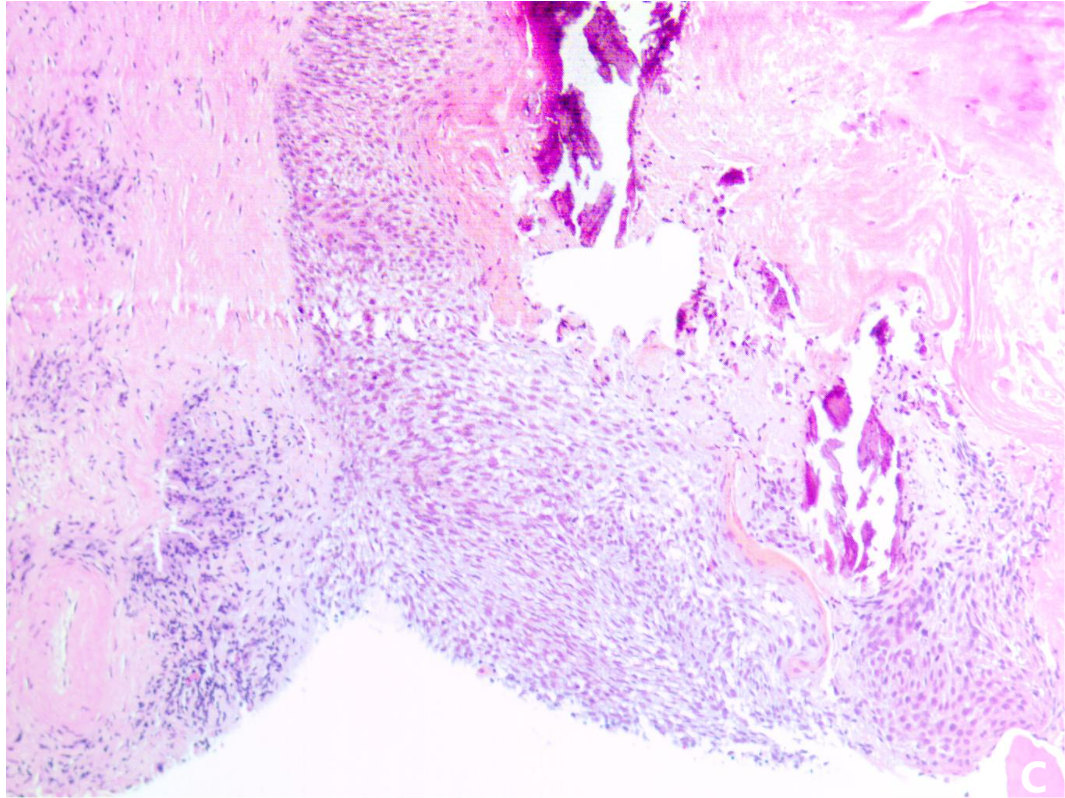
**Supplementary Figure 1.** Figure A、B Enhanced CT：Solid cystic lesions in the right kidney with multiple calcifications.Figure C、DRenal contrast enhanced MR: cystic solid mass in the right upper pole, mixed signal in solid part, limited diffusion in part, nodular log T1 end T2 calcification signa


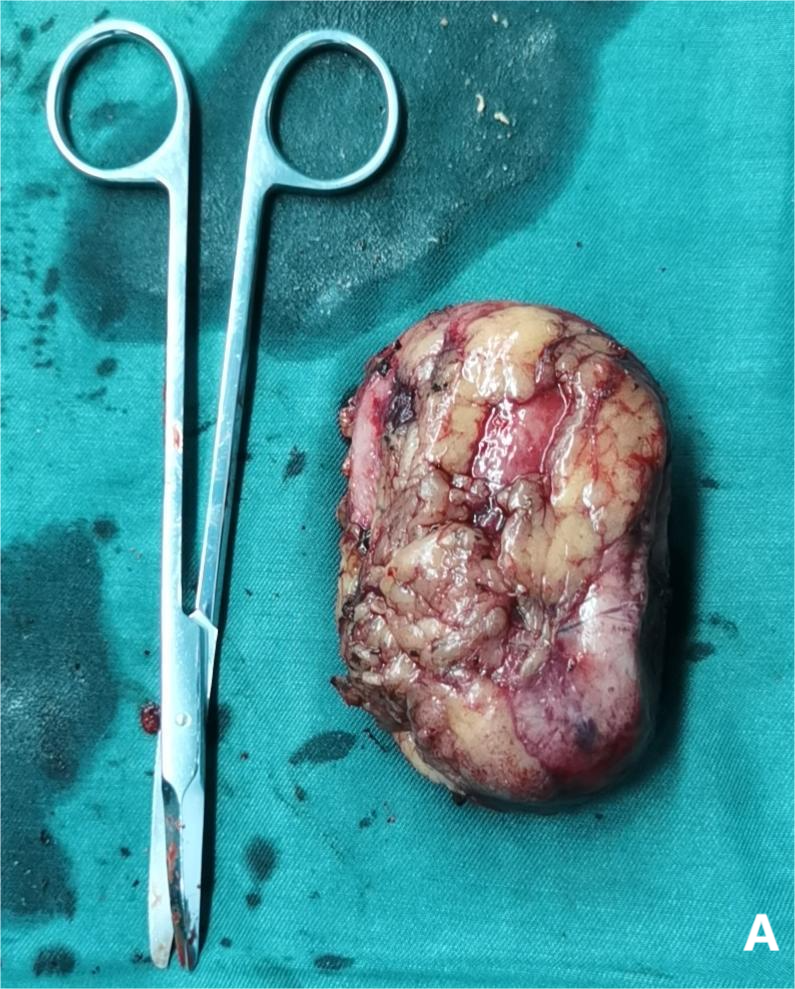


**Supplementary Figure 2.** Figure A: the capsule of the tumor was intact, with a size of 8.5cm * 6cm;Figure B: a large number of "fish like" tissues were found in the tumor capsule;Figure C: high score in pathologySquamous cell carcinoma (he *100).
